# Adrenocorticotrophic hormone secreting pheochromocytoma

**DOI:** 10.4103/0970-1591.60459

**Published:** 2010

**Authors:** Meyyappan M. Ramasamy, Rajaraman Thiagarajan, Pravin S. Dass

**Affiliations:** Department of Urology, Madras Medical College, Chennai - 600 003, India

**Keywords:** Adrenocorticotrophic hormone, Cushing syndrome, pheochromocytoma

## Abstract

We report a rare case of pheochromocytoma of the adrenal gland presenting as Cushing syndrome. A 30 year old woman presented with pain in the left loin and classical Cushingoid features. She was a diabetic and hypertensive on treatment. Magnetic resonance imaging of the abdomen revealed a 3.5×3 cm mass in the left adrenal gland. Urine analysis revealed an elevated 24 hr urinary cortisol level. Clinical diagnosis was Cushing syndrome due to functioning left adrenal tumor, and hence left adrenalectomy was performed laparoscopically. Histopathological examination revealed adrenal pheochromocytoma. Immunohistochemical analysis of the tumor was positive for adrenocorticotrophic hormone and chromogranin.

## INTRODUCTION

Cushing syndrome may be due to ectopic secretion of adrenocorticotrophic hormone (ACTH) from bronchial carcinoid, islet cell tumor, small cell lung carcinoma, medullary carcinoma of the thyroid, thymic carcinoid, disseminated neuroendocrine tumors and disseminated gastrointestinal carcinoid. Ectopic secretion of ACTH may present with features of hypercortisolism similar to pituitary Cushing disease. Cushing syndrome due to ACTH-secreting pheochromocytomas have sporadically been reported in the literature and accounts for 3% of ectopic ACTH-dependent Cushing syndrome.[1] We are reporting a case of ACTH-secreting pheochromocytoma presenting as Cushing syndrome.

## CASE REPORT

**Figure 1:**
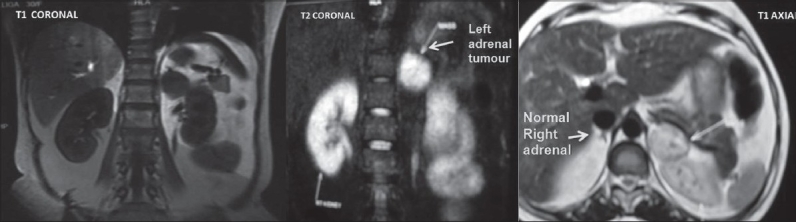
Magnetic resonance imaging of the abdomen-T1 and T2 weighted images showing a 3.5×3 cm, mixed intense mass in the left adrenal gland. Contralateral adrenal gland was normal

**Figure 2:**
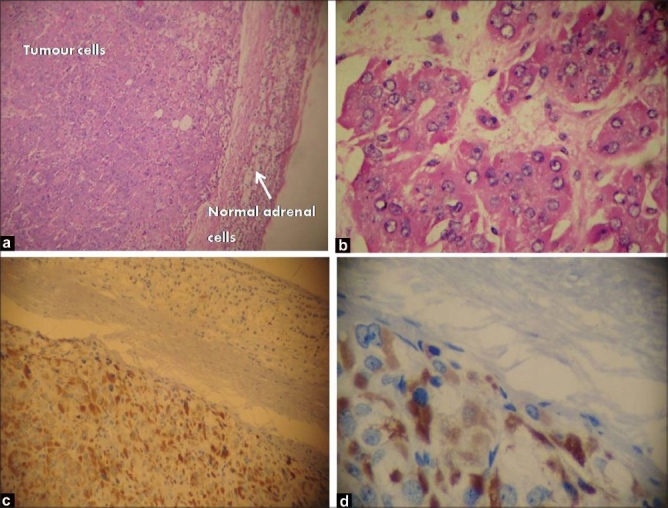
(a) H and E staining (low power view) showing normal adrenal parenchyma with neoplastic cells; (b) High power view of tumor cells arranged in nests, round to polygonal cells with scanty cytoplasm with vesicular nuclei and prominent nucleoli; (c) and (d) Low power and high power views of immunohistochemical staining showing tumor cells stained positive (brown) for adrenocorticotrophic hormone

A 30 year old woman presented to us with a history of left loin pain for 9 months, associated with progressive weight gain. She was diagnosed with diabetes and hypertension three months earlier and started on treatment. She was monoparous and her menstrual cycles were normal. Physical examination revealed typical cushingoid features of centripetal obesity, moon face, buffalo hump, abdominal striae, excessive hair growth and ecchymotic patches in the forearm. Her blood pressure was 160/90 mmHg in the right upper limb, in supine position. Abdominal sonography revealed a 3.5×3 cm mixed echogenic mass involving the left suprarenal gland. Serum cortisol values at 8 am and 4 pm were 39.50 and 24.9 µgm/dl, respectively (normal value 6.2-19.4). A 24 h urinary cortisol level was 540.5 µgm (28.5-213.7). Serum adrenocorticotrophic hormone was 62 pgm/ml (7.2-63.3). A 24 h urine analysis for metanephrines was 1.1 µgm (<1.2) and vanillyl mandelic acid was 12.8 mg (13.6). Magnetic resonance imaging of the abdomen showed a 3.5×3 cm mass in the left adrenal gland. The mass was mixed intense in T1 and T2 weighted images. Contralateral adrenal gland was normal [Figure 1]. With the clinical features, biochemical evidence of hypercortisolism and normal urinary catecholamines, she was diagnosed to have a functioning left adrenal tumor with Cushing syndrome. Laparoscopic adrenalectomy was performed on this patient. No intraoperative hemodynamic fluctuations were noted during anesthesia and surgery, and the postoperative period was uneventful. Cut section of the tumor showed homogenous, smooth, tanned surface with no areas of hemorrhage or necrosis. On the second postoperative day, the patient had profound hypotension. She was managed initially with an injection of hydrocortisone and later switched over to oral steroids. Histopathological examination revealed an encapsulated neoplasm with sheets of polygonal cells containing granular amphophilic cytoplasm and large nuclei, with organoid pattern of arrangement of cells suggestive of pheochromocytoma of the adrenal gland. The adrenal cortex was found to be compressed by the tumor cells with no evidence of adrenocortical hyperplasia. Further immunohistochemical analysis revealed tumor cells staining positive for both ACTH and chromogranin [Figure 2a–d]. At the two month follow up her serum cortisol was 10 µgm/dl, blood pressure was 130/80 mmHg without any antihypertensives.

## DISCUSSION

Ectopic ACTH-dependent Cushing accounts for 10%–15% of all cases of Cushing syndrome.[2] Adrenal pheochromocytoma is a rare cause of ectopic ACTH secretion.[3,4] Cushing syndrome due to ACTH-secreting pheochromocytoma was first described by Meloni in 1966.[5] Pheochromocytoma secretes various substances such as (DOPA, dopamine, ACTH, somatostatin, serotonin, enkephalins, calcitonin, vasoactive intestinal peptide, neuropeptide, lipotropin, β-endorphin), in addition to catecholamines. The symptoms manifested by the patient depend on the predominant secretory product secreted from the tumor. ACTH released from the tumor stimulates the normal adrenal cortex to hypersecrete cortisol. In the present case, the clinical manifestation of the patient was that of Cushing syndrome. Pheochromocytoma was not suspected preoperatively as the patient had no classical paroxysmal spells of hypertension, tachycardia or sweating and her urinary metanephrine was within normal limits. Hence, extensive preoperative preparation with alpha blockers was not done. Further, there were no intraoperative hemodynamic fluctuations such as catecholamine crises. Final diagnosis was made by immunohistochemical analysis, which stained the tumor cells positive for ACTH and chromogranin. Therefore the tumor was found to secrete ACTH predominantly with less catecholamines, explaining the presentation as Cushing syndrome. Pituitary Cushing syndrome can be differentiated from ectopic ACTH secretion by a high-dose dexamethasone suppression test. Despite the unusual presentation of pheochromocytoma as Cushing syndrome, the prognosis is similar to the conventional pheochromocytomas.[6] After removal of the ACTH-secreting adrenal tumor, the patient develops transient hypocortisolism. This is explained by the feedback inhibition of the cortisol-secreting tumor on ACTH secretion from the pituitary. After adrenalectomy, the pituitary recovers from the feedback inhibition and normal cortisol level is restored.

## CONCLUSIONS

Cushing syndrome can be a rare presentation of pheochromocytoma as a result of ectopic secretion of ACTH. It is often difficult to diagnose this presentation preoperatively, especially in cases with a predominant secretion of ACTH rather than catecholamines. In such cases, the final diagnosis can be made only by immunohistochemical analysis.
